# Natural Product Mediated Regulation of Death Receptors and Intracellular Machinery: Fresh from the Pipeline about TRAIL-Mediated Signaling and Natural TRAIL Sensitizers

**DOI:** 10.3390/ijms20082010

**Published:** 2019-04-24

**Authors:** Durray Shahwar, Muhammad Javed Iqbal, Mehr-un Nisa, Milica Todorovska, Rukset Attar, Uteuliyev Yerzhan Sabitaliyevich, Ammad Ahmad Farooqi, Aamir Ahmad, Baojun Xu

**Affiliations:** 1 Laboratory for Translational Oncology and Personalized Medicine, Rashid Latif Medical College, Lahore 54840, Pakistan; durrayshahwar.kh@gmail.com (D.S.); ammadfarooqi@rlmclahore.com (A.A.F.); 2 Department of Biochemistry and Biotechnology, University of Gujrat, Gujrat 34120, Pakistan; m.javediqbal@uog.edu.pk; 3 Department of Biotechnology, University of Sialkot, Sialkot 51310, Pakistan; 4 National Institute of Food Science and Technology, UAF, Faisalabad 38000, Pakistan; mehru912@gmail.com; 5 Department of Pharmacy, Medical Faculty, University of Niš, Dr Zorana Djindjica Boulevard 81, 18000 Niš, Serbia; mimatod@gmail.com; 6 Department of Obstetrics and Gynecology, Yeditepe University, Ataşehir/İstanbul 34755, Turkey; ruksetattar@hotmail.com; 7 Kazakhstan Medical University “KSPH”, Almaty 050060, Kazakhstan; e.uteuliyev@ksph.kz; 8 Department of Oncologic Sciences, Mitchell Cancer Institute, University of South Alabama, Mobile, AL 36604, USA; aahmad@health.southalabama.edu; 9 Food Science and Technology Program, Beijing Normal University-Hong Kong Baptist University United International College, Zhuhai 519087, Guangdong, China

**Keywords:** apoptosis, cancer, death receptors, natural products, TRAIL

## Abstract

Rapidly developing resistance against different therapeutics is a major stumbling block in the standardization of therapy. Tumor necrosis factor (TNF)-related apoptosis-inducing ligand (TRAIL)-mediated signaling has emerged as one of the most highly and extensively studied signal transduction cascade that induces apoptosis in cancer cells. Rapidly emerging cutting-edge research has helped us to develop a better understanding of the signaling machinery involved in inducing apoptotic cell death. However, excitingly, cancer cells develop resistance against TRAIL-induced apoptosis through different modes. Loss of cell surface expression of TRAIL receptors and imbalance of stoichiometric ratios of pro- and anti-apoptotic proteins play instrumental roles in rewiring the machinery of cancer cells to develop resistance against TRAIL-based therapeutics. Natural products have shown excellent potential to restore apoptosis in TRAIL-resistant cancer cell lines and in mice xenografted with TRAIL-resistant cancer cells. Significantly refined information has previously been added and continues to enrich the existing pool of knowledge related to the natural-product-mediated upregulation of death receptors, rebalancing of pro- and anti-apoptotic proteins in different cancers. In this mini review, we will set spotlight on the most recently published high-impact research related to underlying mechanisms of TRAIL resistance and how these deregulations can be targeted by natural products to restore TRAIL-mediated apoptosis in different cancers.

## 1. Introduction

Cancer is a multifaceted and therapeutically challenging disease. Groundbreaking discoveries in the past few decades have enabled us to develop a sharper understanding of intra- and inter-tumor heterogeneity, loss of apoptosis, oncogenic overexpression, inactivation of tumor suppressors, and deregulation of spatio-temporally controlled signal transduction cascades which play a central role in cancer onset and progression [1,2,3,4]. Loss of apoptosis has been, and still remains, a subject of intense discussion for basic and clinical scientists. Cutting-edge research has sequentially revealed that the inhibition of cell death in combination with mitogenic oncogenes promoted cancer in animal models [5]. There were some exciting developments which highlighted that many oncogenic pathways inhibited apoptosis, whereas tumor suppressors (e.g., p53) played an instrumental role in the induction of apoptosis [6]. More importantly, approval of BH3 (BCL-2 homology domain-3) mimetics by the Food and Drug Administration (FDA) for the treatment of 17p-deleted CLL is a milestone in the field of molecular therapy. Therefore, researchers have focused on the identification of pathways and proteins that can efficiently induce apoptosis and simultaneously induce regression in xenografted mice. There has always been a quest to identify molecules that can cause maximum damage to cancer cells while leaving normal cells intact. In accordance with this concept, TRAIL (TNF-related apoptosis-inducing ligand) has emerged as a scientifically approved protein that can induce apoptosis, specifically in cancer cells. Initial findings reported by researchers were tremendously encouraging, and urged cotemporary scientists to further dissect this intriguing and therapeutically important pathway. Consequently, substantial excitement encompasses the premium potential of natural products to effectively restore apoptosis in TRAIL-resistant cancers. 

There are some detailed reviews about TRAIL-mediated signaling in different cancers, but we have exclusively focused on the most recent evidence related to positive and negative regulators of TRAIL-mediated signaling in this review. We have also summarized how different natural products effectively restored apoptosis in TRAIL-resistant cancers. Before providing an overview of the natural product-mediated regulation of the TRAIL-driven pathway, we will discuss some of the most important advancements in the TRAIL-mediated signaling pathway. 

## 2. Molecular Insights of TNF-Related Apoptosis-Inducing Ligand (TRAIL)-Mediated Signaling

**Figure 1:**
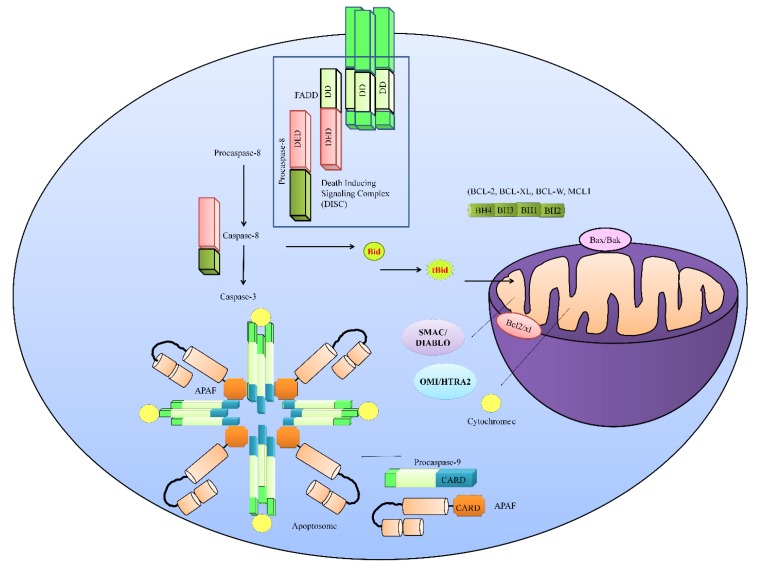
TRAIL (TNF-related apoptosis-inducing ligand)-mediated signaling. TRAIL transduces the signals intracellularly through death receptors. Death-inducible signaling complex (DISC) is formed by the interaction of the death receptor Fas-associated protein with death domain (FADD) and pro-caspase-8. Formation of DISC is necessary for the activation of caspase-8. Caspase-8 activates its downstream effector caspase-3. However, caspase-8 may also proteolytically process Bid to initialize the intrinsic pathway. The intrinsic pathway is triggered following entry of truncated Bid into mitochondria. Cytochrome c and SMAC are released from mitochondria and an apoptosome was formed in the cytoplasm by the assembly of apoptotic protease activating factor (APAF), cytochrome c, and pro-caspase-9. The apoptosome is necessary for the activation of caspase-9 and it can further activate caspase-3 to induce apoptosis in cancer cells. In healthy cells, APAF is present as an autoinhibitory monomer. However, mitochondrial outer membrane permeabilization (MOMP) and subsequent release of cytochrome c unlocks APAF.

Overwhelmingly, increasing and continuously upgrading discoveries in the field of apoptotic cell death have enabled us to develop a better knowledge of this area. Researchers have extensively characterized two primary death-signaling cascades, the extrinsic and intrinsic pathways. When the signaling is “switched on”, it results in the activation of downstream effector molecules for both pathways that mediate apoptosis. Caspases belong to a group of cysteine proteases which proteolytically process a variety of cytoplasmic and nuclear substrates [7]. The extrinsic, or death -receptor-mediated, pathway is activated and functionalized through the binding of death ligands such as TRAIL, TNFα (tumor necrosis factor α) and Fas ligand (FasL) to specific receptors (e.g., TRAILR1/DR4, TRAIL2/DR5, TNFR, Fas). Ligand–receptor interaction results in the recruitment of the cytoplasmic adaptor protein FADD (Fas-associated protein with death domain) to death domains present in the cytoplasmic segment of the death receptor (shown in Figure 1). Death domains present in death receptors served as recruiting modules and heterodimerized with the death domains of client proteins. FADD contains death domains that can link the death receptor to procaspase-8 to form a death-inducing signaling complex (DISC). 

Activation of the mitochondrial or intrinsic pathway is induced either through radiation or chemotherapy. Caspase-8-mediated truncation of Bid also played a dominant role in activating the mitochondrially driven pathway. Translocation of truncated Bid into mitochondria caused mitochondrial permeabilization and release of apoptogenic proteins, including cytochrome c and second mitochondrial-derived activator of caspases (SMAC) from the mitochondria into the cytosol. Cytosolic cytochrome c interacted with apoptotic protease activating factor-1 (APAF1) and formed a multimeric complex termed the apoptosome (shown in Figure 1). The apoptosome recruited, cleaved, and activated caspase-9 and -3. SMAC promoted apoptosis by binding to and degrading multiple inhibitors of apoptotic proteins (IAPs): XIAP, c-IAP1, and c-IAP2. Therefore, it seems clear that TRAIL-mediated signaling following the activation of caspase-8 is dichotomously branched. Either caspase-8 can activate its downstream effector caspases or it can proteolytically process the Bid protein to initialize the intrinsic pathway that routes through mitochondria. It appears to be important that the TRAIL-induced intracellular signaling pathway is not as simple as previously surmised. A wealth of information points towards myriad signaling pathways which crosstalk with different proteins of the TRAIL-mediated signaling pathway, and play a critical role. Therefore, we partitioned our review into negative and positive regulators of TRAIL-mediated signaling to comprehensively analyze the most recent breakthroughs made in uncovering mechanisms that inhibit or potentiate TRAIL-triggered apoptotic cell death. 

## 3. Negative Regulators of TRAIL-Mediated Signaling

Because TRAIL-based therapies have entered into various phases of clinical trials, it is essential to drill down deep into TRAIL-mediated signaling and further unfold the mysterious aspects of this pathway. The overexpression and stabilization of anti-apoptotic proteins is necessary to induce resistance against TRAIL. Various lines of evidence have revealed that cancer cells developed resistance against TRAIL mainly through the overexpression and stabilization of anti-apoptotic proteins. We have partitioned this section into modes and strategies used by cancer cells to develop resistance against TRAIL. (1) Stabilization of anti-apoptotic proteins; (2) Noncoding RNAs also play their role in rewiring cell-signaling pathways to potentiate TRAIL resistance; (3) Ubiquitination is a very critical mechanism in the context of anti-apoptotic and pro-apoptotic protein degradations. It has been seen that pro-apoptotic proteins are degraded but anti-apoptotic proteins escape from these death-tagging molecules in TRAIL-resistant cancer cells; (4) Recent discoveries have unmasked unique locations and functionalities of death receptors. Death receptors not only activate classical pathways to induce apoptosis, but their movement has also been tracked in the nucleus to modulate miRNA biogenesis. Therefore, we will discuss these interesting topics in the upcoming section and try to critically evaluate their implications. 

**Figure 2:**
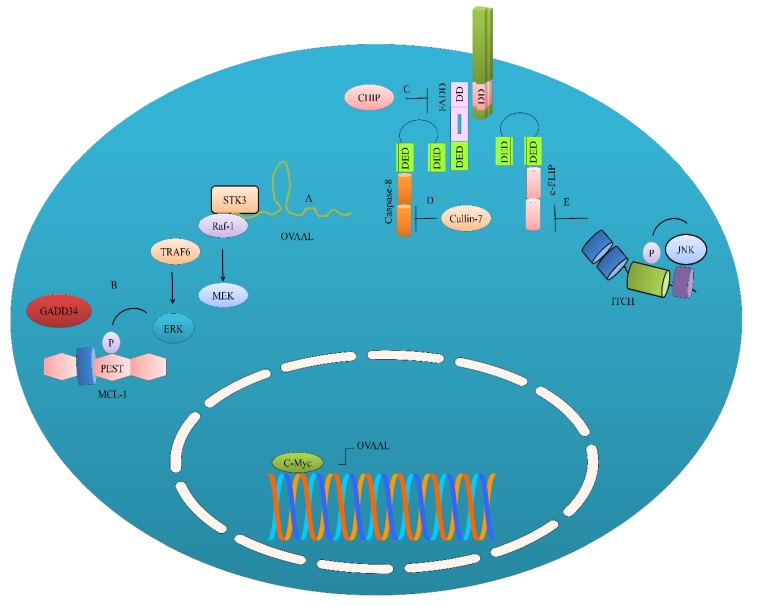
Negative regulation of the TRAIL-driven pathway. (**A**) OVAAL, a long non-coding RNA, has been shown to promote interaction between STK3 and Raf-1. STK3 and Raf-1 work synchronously and activate the MEK/ERK pathway; (**B**) ERK has also been shown to stabilize MCL-1. ERK is also activated by TRAF6 and GADD34 to stabilize MCL-1. OVAAL is transcriptionally upregulated by c-Myc; (**C**) Post-translational modifications have been shown to effectively regulate caspase-8 and FADD. FADD is negatively regulated by C terminus HSC70-interacting protein (CHIP); (**D**) Caspase-8 is negatively regulated by Cullin-7; (**E**) ITCH involved in the negative regulation of c-FLIP.

GADD34 (growth arrest and DNA damage-inducible protein 34) overexpression resulted in an increase in MCL-1 levels. Knockdown of GADD34 resulted in marked reduction in MCL-1 levels in both SMMC-7721 and HepG2 cells [8]. GADD34 suppressed proteasomal degradation of MCL-1 mainly through promoting extracellular signal-regulated kinase (ERK1/2)-mediated phosphorylation of proline (P), glutamic acid (E), serine (S), and threonine (T) (PEST) domains in MCL-1 (shown in Figure 2). TNF receptor associated factor 6 (TRAF6) played a central role in directing ERK1/2 phosphorylation and the enhancement of the stability of MCL-1 levels in HepG2 cells. GADD34 knockdown exerted repressive effects on the levels of MCL-1 in SMMC-7721 and HepG2 cells. Furthermore, TRAF6 knockdown also enhanced TRAIL-mediated apoptosis, as evidenced by considerably reduced levels of p-ERK1/2 and MCL-1 [8].

CIB1 (calcium and integrin-binding protein 1) played a contributory role in the development of resistance against chemotherapeutics and TRAIL [9]. Intriguingly, expression levels of DR5 were noted to be dramatically enhanced in CIB1-depleted MDA-436 breast cancer cells [9]. These findings appear to be exciting, but scientists have not provided a comprehensive pathway opted by CIB1 to inhibit DR5. There is an urgent need to put the missing pieces of information together to uncover the underlying mechanisms which inhibit, repress, or degrade death receptors. 

Ovarian adenocarcinoma-amplified lncRNA (OVAAL) has been shown to play an instrumental role in the development of resistance against TRAIL [10]. OVAAL interacted with STK3 (serine/threonine-protein kinase-3), which consequently enhanced the structural association between Raf-1 and STK3. Studies have shown that the ternary complex OVAAL/STK3/Raf-1 activated rapidly accelerated fibrosarcoma/mitogen-activated protein kinase kinase (RAF/MEK/ERK signaling pathway and promoted Mcl-1-mediated survival and c-Myc-driven proliferation of the ME4405 and HCT116 cells. c-Myc has also been noted to transcriptionally upregulate OVAAL (Figure 2). However, expectedly, TRAIL-mediated apoptosis was considerably enhanced in OVAAL-silenced ME4405 cells [10]. These findings clearly indicate that non-coding RNAs stabilize anti-apoptotic proteins via the rewiring of signaling pathways. 

Detailed mechanistic insights revealed that cytoplasmic PARP-1 was recruited into the TRA-8-activated DISC and sustained Src-mediated pro-survival signals [11]. However, the knockdown of PARP-1 not only interfered with the activation of Src but also improved TRA-8-mediated apoptotic cell death in BxPc-3 and MiaPaCa-2 cells [11]. 

Cullin-7 has recently been shown to physically interact with caspase-8 [12]. CUL7 prevented the activation of caspase-8 by promoting post-translational modifications of caspase-8 by the addition of non-degradative polyubiquitin chains at the 215th lysine (shown in Figure 2). Knockdown of CUL7 re-sensitized cancer cells to TRAIL-triggered apoptotic cell death. Tumor growth was significantly inhibited in mice xenografted with CUL7-silenced MDA-MB-231 cells [12]. CHIP (C terminus HSC70-interacting protein) induced the K6-linked polyubiquitylation of FADD and suppressed the formation of the DISC (Figure 2) [13].

Monocyte chemotactic protein-induced protein-1 (MCPIP1), a deubiquitinating enzyme, promoted the lysosomal degradation of DR5 [14]. MCPIP1 knockdown facilitated DISC formation [14]. At a more basic level, it would be extremely interesting to see how different proteins sort death receptors as well as pro-apoptotic and anti-apoptotic proteins for degradation in different cancers. 

Karyopherin β1 (KPNB1) played an instrumental role in the nuclear import of DR5. KPNB1 transported DR5 into the nucleus, while inhibition of KPNB1 restored DR5 levels on the cell surface of glioblastoma cells [15]. These findings are exciting, and it needs to be seen how DR5 behaves in the nucleus. Certain hints have emerged which have scratched the surface of regulatory role of DR5 in the biogenesis of microRNAs. Nuclear DR5 inhibited the maturation of a miRNA, let-7, in pancreatic cancer cells and increased their proliferation abilities [15]. Astonishingly, two functional nuclear localization signal (NLS) sequences have previously been identified in DR5 [16]. Additionally, it was shown that importin-β1 interacted with DR5 and shipped it to the nucleus in HeLa cells [16]. It will not be invalid if we say that shuttling of death receptors in the nucleus is the “tip of the iceberg” and needs detailed and in-depth research. At the moment, we have a segmented view about the internalization of death receptors from the cell surface and “moonlight activity” of death receptors in the nucleus. 

It has recently been convincingly revealed that circulating tumor cells (CTCs) demonstrated rapid autophagic flux, characterized by an accumulation of autophagosome organelles [17]. Notably, there was substantial evidence highlighting the presence of DR5 in the autophagosomes, followed by degradation by lysosomes [17]. Overall, these findings clearly suggest that CTCs escape from TRAIL-mediated killing activities by reducing the cell surface expression of DR5.

## 4. Positive Regulators of TRAIL-Mediated Signaling

RUNX3 (RUNT-related transcription factor-3) played a central role in stimulating the expression of DR5 [18]. DR5 was markedly increased in RUNX3-overexpressing HT29 cells. RUNX3-mediated reactive oxygen species (ROS) can lead to an enhanced ER stress in cancer cells. Therefore, the overexpression of RUNX3 induced DR5 via IRE1α-JNK-CHOP pathway. Treatment of the cells with an ROS scavenging chemical, *N*-acetyl-l-cysteine, severely compromised TRAIL-mediated apoptosis in RUNX3-overexpressing cancer cells [18]. Superoxide dismutases (SODs) constitute the antioxidant defense grid. RUNX3 has been shown to transcriptionally repress SOD3 to induce ROS generation and upregulate DR5. Mechanistically it has been shown that RUNX3 occupied RUNX3 binding sites present within the promoter region of SOD3 and inhibited its transcription [18]. The findings of this study are exciting, and future studies must converge on the analysis of the role of RUNX3 in different cancers. It will be informative to see if RUNX3 is functionally active in other TRAIL-resistant cancers. 

Fucosylation is a post-translational modification of critical importance that plays a crucial role in improving TRAIL-mediated apoptosis [19]. Stably and transiently overexpressed FUT3 and FUT6 dramatically enhanced TRAIL-mediated apoptosis in HCT116 and DLD-1 cells. Activation and cleavage mechanisms of caspase-8 and PARP-1 were noted to be more pronounced in cells overexpressing FUT6 and FUT3. More importantly, a significantly higher fraction of signalosomes was noticed, as evidenced by highly increased DISC-associated caspase-8 complexes in FUT3-overexpressing cells [19]. Harakiri (HRK), a BH3-only protein of the Bcl-2 family, has been shown to promote TRAIL-mediated apoptosis in glioblastoma cells [20].

ITCH, a homologous to the E6AP carboxyl terminus (HECT) domain E3 ligase, is reportedly involved in the negative regulation of c-FLIP [21]. JNK phosphorylation sites have been mapped in the protein sequence of ITCH. Levels of phosphorylated ITCH (p-ITCH) were found to be enhanced in MCF-7- and T47D-derived tamoxifen-resistant and faslodex-resistant cells. Moreover, inhibition of JNK resulted in the inactivation of ITCH and reduced p-ITCH levels, and consequently c-FLIP levels were restored in cancer cells [21,22]. Therefore, in future studies it will be paramount to investigate if additional kinases are involved in the activation of ITCH and if ITCH can post-translationally modify various other negative regulators of apoptosis in different cancers. 

## 5. Natural-Product-Mediated Restoration of TRAIL-Mediated Apoptosis in Different Cancers

Natural products have captivated tremendous attention because of their premium pharmacological properties. There has been a longstanding quest to identify products that can be combined with TRAIL to maximize the apoptosis in TRAIL-resistant cancers. Therefore, in this specific section, we provide an update about products obtained from natural sources which can restore apoptosis in resistant cancer cells. 

Auriculasin, a prenylated isoflavone, was highly effective when used in combination with TRAIL [23]. Auriculasin and TRAIL combinatorially increased the expression of Bax, AIF, endo G, and cytochrome c. Auriculasin triggered the upregulation of DR5 but levels of DR4 remained unchanged [23]. Cannabidiol, pharmacologically potent cannabinoid isolated from the *Cannabis* plant, induced the upregulation of the protein and cell surface expression of TRAIL-R2/DR5 [24]. 

Andrographolide isolated from *Andrographis paniculata* enhanced the expression of DR4 and DR5 mainly through increasing the levels of p53 [25]. As expected, andrographolide-mediated upregulation of DR4 and DR5 was not observed in p53 knockdown T24 cells [25]. 

Periplocin obtained from Cortex Periplocae led to a dose-dependent enhancement of the expression levels of DR4 and DR5 on the surface of MGC-803 and SGC-7901 cells [26]. Periplocin and TRAIL combinatorially enhanced the levels of p-ERK1/2 and EGR1 (early growth response-1). However, as expected, treatment with an inhibitor of MEK (PD98059) severely interfered with the upregulation of DR4 and DR5 in cancer cells. EGR1 overexpression induced the stimulation of DR4 and DR5, while EGR1 knockdown exerted repressive effects on the expression levels of DR4 and DR5 [26]. Weights and volumes of tumors from mice treated combinatorially with TRAIL and periplocin were found to be significantly reduced as compared to mice treated with periplocin or TRAIL alone [26].

**Figure 3:**
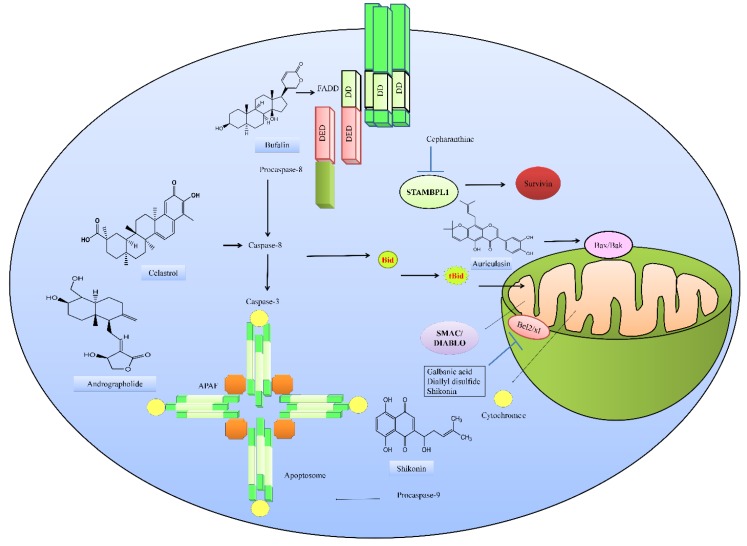
Natural-product-mediated multi-step regulation of the TRAIL-driven pathway.

In combination with TRAIL, the sesquiterpene coumarin galbanic acid, worked effectively against non-small-cell lung cancer cells [27]. Galbanic acid and TRAIL induced the upregulation of DR5 and simultaneously suppressed DcR1. Galbanic acid and TRAIL attenuated the expression of Bcl-2 (B-cell lymphoma-2), Bcl-xL (B-cell lymphoma-extra-large) and XIAP (X-linked inhibitor of apoptotic proteins) in H460/R cells (Figure 3) [28].

Diallyl disulfide (DADS) and TRAIL jointly repressed Bcl-2 in colorectal cancer cells [28]. Bufalin, a cardiotonic steroid isolated from the secretion of parotid glands and skin of Chansu and black-spectacled toad has recently been shown to restore apoptosis in TRAIL-resistant cancer cells by the regulation of pro- and anti-apoptotic proteins [29]. Bufalin increased ER stress associated proteins (GRP78, caspase-4, IRE-1α, IRE-1β, ATF-6α, GADD153, and Calpain 1). Bufalin increased Bax; cytochrome c; caspase-3, -8, and -9; AIF; and Endo G, but simultaneously reduced Bcl-2 in NPC-TW 076 cells. Furthermore, bufalin elevated the expression levels of TRAIL, FADD, DR4, and DR5 [29].

Shikonin is a medicinally important product isolated from the root of *Lithospermum erythrorhizon* that was shown to work effectively with TRAIL against A549 cells [30]. Shikonin and TRAIL synergistically reduced Mcl-1, Bcl-2, Bcl-xL, XIAP, and c-FLIP and upregulated the levels of Bid (Figure 3) [30]. Celastrol, a triterpenoid isolated from *Tripterygium wilfordii* worked effectively with TRAIL and inhibited autophagic influx in A549 cells [31]. 

Remarkable advancements have been made in the molecular biology of autophagy, and scientists are focusing on solving questions regarding how this pathway can be harnessed to improve clinical outcomes [32,33]. In 2016 the scientific community acknowledged the true potential of autophagy in health and disease, and Yoshinori Ohsumi was awarded the Nobel Prize for Physiology or Medicine for his outstanding work, which elevated our understanding about intricate mechanisms of autophagy to the next level. Autophagy plays a central role in the maintenance of homeostasis. Autophagy begins when double-membrane autophagosomes engulf fractions of the cytoplasm, which is followed by the fusion of these vesicles with lysosomes, and autophagic contents are consequently degraded [33,34]. Phosphatidylethanolamine (PE) is conjugated to cytoplasmic LC3I to generate the lipidated form, LC3II, and consequently LC3II is incorporated into the growing membrane [34].

Celastrol treatment induced an increase in the levels of LC3II and p62. However, authors did not report the effects induced by combinatorial treatment with celastrol and TRAIL [31]. It was only suggested that celastrol and TRAIL synergistically enhanced ROS generation in cancer cells. Various other natural products, particularly 6-shogaol, were also found to increase the levels of LC3II and p62 in Huh7 cells [35].

2-Deoxy-d-glucose potentiated TRAIL-triggered apoptotic cell death, in part through suppressing JNK-mediated cytoprotective autophagic signaling in SGC7901 and MGC803 cells [36]. Chloroquine and TRAIL synergistically enhanced LC3II levels in pancreatic cancer cells [37].

Excitingly, flow cytometry analyses revealed that toosendanin induced a reduction of membrane DR5 [38]. However, these effects were prevented by inhibitors of autophagy (3-methyladenine). 3-Methyladenine increased the basal level of membrane DR5, which clearly indicated that autophagy centrally regulated the membrane distribution of DR5 [38,39].

It is important to mention that autophagy has a dualistic role in TRAIL-mediated apoptosis. There is also sufficient evidence related to the positive regulation of TRAIL-mediated apoptosis by autophagy. Different natural products have also been shown to potentiate TRAIL-mediated apoptosis through the induction of autophagy. Ginsenosides are biologically active constituents of ginseng, and potentiate TRAIL-mediated apoptosis through the induction of autophagy [40]. Juglanin also induced autophagy and consequently enhanced TRAIL-mediated apoptosis in cancer cells. Juglanin induced the regression of tumors in mice subcutaneously injected with A549 cells [41]. 

Ursolic acid stimulated the expression of DR4 and DR5 and simultaneously downregulated c-FLIP_L_ and re-sensitized TRAIL-resistant triple-negative breast cancer cells to apoptosis [42]. 

Cepharanthine, a biscoclaurine alkaloid isolated from *Stephania cepharantha*, has been shown to be effective against renal carcinoma cells [43]. Cepharanthine time-dependently induced the downregulation of survivin protein levels. It has been mechanistically demonstrated that cepharanthine promoted c-FLIP degradation. It was observed that use of proteasome inhibitor reversed cepharanthine-induced c-FLIP degradation. STAMBPL1 is one of the JAB1/MPN/Mov34 metalloenzymes (JAMM) deubiquitin enzymes, and is reportedly involved in the regulation of different processes. Cepharanthine dose-dependently downregulated STAMBPL1 and increased USP53 expression. Ectopic expression of STAMBPL1 significantly inhibited cepharanthine-induced reduction in the levels of survivin [43]. Overall, these findings clearly suggest that cepharanthine enhances TRAIL-induced apoptosis by promoting the degradation of survivin through STAMBPL1 downregulation in renal carcinoma cells (Figure 3).

C-27-carboxylated oleanolic acid derivatives have been shown to stimulate the expression of DR5 through CHOP [44]. Treatment of glioma U251MG and LN428 cells with 3β-hydroxyolean-12-en-27-oic acid (C27OA-1) induced an increase in the expression of CHOP. C27OA-1 considerably activated p38 and ERK (extracellular signal regulated kinase). Treatment of U251MG cells with p38 inhibitors (SB203580) severely abrogated C27OA-1-mediated increase in expression levels of CHOP and DR5 [44]. 

Lambertianic acid is a biologically active product isolated from *Pinus koraiensis* that was efficient against non-small-cell lung cancer. Lambertianic acid and TRAIL upregulated DR4. Furthermore, lambertianic acid and TRAIL markedly reduced p-NF-κB, p-IκB, and c-FLIP in A549 and H1299 cells [45]. 

## 6. Concluding Remarks

The cancer genome atlas (TCGA) network groups have comprehensively reported the genomic landscape for over 30 different cancer types [46]. More importantly, many of these malignancies have a subset of cases which harbored genomic alterations in components of extrinsic or intrinsic pathways, including overexpression and amplification of FADD and IAP (inhibitor of apoptotic proteins), as well as the identification of mutations in caspase-encoding genes [47,48]. 

It seems surprising to note that although scientists have uncovered tremendous information about the TRAIL-mediated signaling pathway, we have not sufficiently investigated the natural-product-mediated regulation of the TRAIL-driven pathway. Even though we have seen that natural products triggered the upregulation of death receptors and induced the re-balancing of pro- and anti-apoptotic proteins, we still have unanswered questions and visible knowledge gaps in our understanding related to the realization of products derived from medicinally important natural sources. 

Different ubiquitin ligases (e.g., MARCH8) have been shown to ubiquitinate DR4 at 273rd lysine and induce degradation [49]. CHIP (C terminus HSC70-interacting protein) induced the K6-linked polyubiquitylation of FADD and suppressed the formation of the DISC [13]. CUL7 prevented the activation of caspase-8 mainly by promoting the modification of caspase-8 with non-degradative polyubiquitin chains [12]. However, the natural-product-mediated targeting of ubiquitin ligases to restore TRAIL-mediated apoptosis is an insufficiently studied area of research. There is a need to focus on the ubiquitin-ligase-targeting abilities of natural products which can later be used effectively in TRAIL-resistant cancers. 

Another important and exciting area of research that needs detailed research is the microRNA regulation of the TRAIL-driven pathway. Rapidly emerging scientific reports have started to shed light on the central role of microRNAs in the modulation of the TRAIL-mediated pathway. Certain pieces of evidence have suggested that maritoclax, isolated from marine bacteria, promoted miR-708-mediated targeting of c-FLIP and restored apoptosis [50]. Interestingly, α-mangostin, a xanthone isolated from the mangosteen fruit, restored apoptotic death in TRAIL-resistant colon cancer DLD-1 cells [51]. Α-Mangostin effectively promoted DR5 oligomerization. Α-Mangostin exerted repressive effects on miR-133b and stimulated the expression of DR5 [51].

However, we have not yet witnessed considerable experimental work related to the natural-product-mediated regulation of miRNAs to restore apoptosis in TRAIL-resistant cancers. Likewise, different xenografted mice model studies are necessary for an effective evaluation of the potential of natural products in inducing tumor regression. 

## Abbreviations

APAF1	apoptotic protease activating factor-1
Bcl-2	B-cell lymphoma-2
Bcl-xL	B-cell lymphoma-extra-large
CHIP	C terminus HSC70-interacting protein
CIB1	calcium and integrin-binding protein 1
CTCs	circulating tumor cells
DISC	death-inducing signaling complex
EGR1	early growth response-1
FADD	Fas-associated protein with death domain
GADD	growth arrest and DNA damage-inducible protein
IAP	inhibitor of apoptotic proteins
OVAAL	ovarian adenocarcinoma-amplified lncRNA
RUNX3	RUNT-related transcription factor-3
SMAC	second mitochondrial-derived activator of caspases
SOD	superoxide dismutase
TNFα	tumor necrosis factor α
TRAIL	TNF-related apoptosis-inducing ligand
XIAP	X-linked inhibitor of apoptotic proteins
